# Viscoelastic and Thermal Properties of Styrene Modified Fir Wood

**DOI:** 10.3390/polym14040786

**Published:** 2022-02-17

**Authors:** Branimir Jambreković, Emi Govorčin Bajsić, Nikola Španić, Tomislav Sedlar, Tomislav Sinković

**Affiliations:** 1 Faculty of Forestry and Wood Technology, University of Zagreb, 10000 Zagreb, Croatia; bjambreko@sumfak.unizg.hr (B.J.); nspanic@sumfak.unizg.hr (N.Š.); tsinkovic@sumfak.unizg.hr (T.S.); 2 Faculty of Chemical Engineering and Technology, University of Zagreb, 10000 Zagreb, Croatia; egovor@fkit.hr

**Keywords:** fir wood (*Abies alba* Mill.), grafting modification surface, chemical modification, FTIR-ATR, DMA, TGA

## Abstract

The modification of wood and its surface is one of the challenges that is being perfected with the aim of transitioning to sustainable management. This study investigated the dynamic mechanical and thermal behaviour of unmodified and styrene modified fir wood (*Abies alba* Mill.). Styrene monomer was chosen and impregnated into the porous structure of fir wood by reversible addition-fragmentation chain transfer (RAFT) polymerisation. Attenuated total reflection Fourier-transform infrared spectroscopy (FTIR-ATR), dynamic mechanical analysis (DMA), and thermogravimetric analysis (TGA) were employed to characterise the chemical structure, viscoelastic properties, and thermal stability of unmodified and modified (surface-modified) wood. All tests have to be regarded as being preliminary due to the small number of specimens. Fourier transform infrared analysis showed evidence of the phenyl group from styrene at 700 cm^−1^. DMA results showed that the modified wood caused an increase in the glass transition temperature relative to the unmodified wood. In addition, modification with styrene improves thermal stability, as revealed by thermogravimetric analysis (TGA).

## 1. Introduction

Wood is one of the most important renewable materials and exhibits superior properties in many cases. Wood is a three-dimensional, highly complex cellular material. It is a biopolymer, which is hygroscopic, viscoelastic, and anisotropic [1]. The wood comprises cellulose, hemicelluloses, and lignin as main components [2], with some inorganics and extractives present as minor components.

Around 40% of the dry weight of wood consists of cellulose. Cellulose is a high-molecular-weight linear polymer built up of D-glucose units linked together by β-(1-4)-glycosidic bonds. The cellulose molecule is linear, and it is therefore capable of forming strong intra- and intermolecular hydrogen bonds and aggregated bundles of molecules [3]. Hemicelluloses are heterogeneous polymers built up of several monomers, such as mannose, arabinose, xylose, galactose, and glucose. 20–30% of the dry weight of wood consists of hemicelluloses. Lignins are complex heterogeneous three-dimensional, amorphous polymers (p-coumaryl alcohol, coniferyl alcohol, and sinapyl alcohol) that constitute approximately 30% of the dry weight of wood [2]. Lignin is often called the cementing agent that binds individual cells together.

Wood has played a significant role throughout human history—it has been used by humanity for millennia due to its numerous advantages. Nowadays, wood is known as a good building and engineering material due to its renewable and economic properties. Besides these properties, wood has several properties which are not appreciated in many cases, such as swelling and shrinkage with varying moisture content, biodegradability, flammability, and degradability by ultraviolet (UV) light, acid, and bases [4,5,6,7]. Wood is a porous material with a complex structure and recognisable by its inhomogeneity and anisotropy. The structure of wood components and the understanding of water bonding at different moisture contents are essential for chemical modification [8].

From a macroscopic point of view, fir is a species with no difference between heartwood and sapwood colour, yellowish-white to greyish-white and reddish-white colour. In the study of wood stability on facades [9], fir was characterised as a type of wood of medium to high water absorption with medium stability to dimensional changes and the area of application in external and internal structure. In case of application in external constructions, fir should be impregnated. Previous research has shown that fir as a wood raw material occupies a significant place in construction along with other types of wood used in construction today and the need for detailed characterisation of fir. DMA measurements in isothermal conditions in the high-temperature range on spruce and beech can be successfully used to study the dynamic mechanical behaviour of wood and its thermo oxidative degradation [10].

Chemical modification of wood has received much attention since the middle of the 20th century. Recently, many studies were conducted on the chemical modification of wood to improve dimensional stability, mechanical properties, durability, and wood surface [11,12,13,14,15]. Grafting hydrophobic monomers to hydroxyl groups in the cell wall increases the hydrophobicity of wood [16]. Vinyl monomer impregnation followed by in situ polymerisation represents a promising way to enhance mechanical, dimensional, and thermal stability as well as fungal and insect resistance of low natural durability wood. Several studies [17,18,19] report newer polymerisation techniques known as “controlled living free-radical polymerisations” (or CRP). The growth, chain transfer, and termination reactions are controlled to yield polymers with a desired molecular weight and narrow polydispersity index (PDI). The two most prevalent techniques are Atom Transfer Radical Polymerization (ATRP) and Reversible Addition-Fragmentation Chain Transfer Polymerization (RAFT) [17,18,19]. ATRP technique is suitable to graft poly (methyl methacrylate) (PMMA), whereas RAFT polymerisation technique is adequate for grafting poly (vinyl alcohol) (PVA), poly(styrene) (PS), and poly (butyl acrylate) (PBA) to wood fibres [20,21].

In this study, fir wood (*Abies alba* Mill.) was modified with styrene monomer via RAFT polymerisation. The effect of modification on the physical and viscoelastic properties and thermal stability was investigated.

## 2. Experimental

### 2.1. Materials

Fir wood (*Abies alba* Mill.) was used for the study. The wood samples with nominal dimensions of 45 mm × 10 mm× 2 mm (l × w × t) were prepared from the fir wood boards cut into lamellae. The length of the test samples corresponded with the longitudinal axis of wood. All wood samples were oven-dried at 103 °C to constant weight and stored in a desiccator over silica gel before treatment. The final average mass of test samples was 0.45 g, with an average moisture content of 7.63%. The moisture content was obtained by the gravimetric method according to ISO 13061-1:2014 [22].

Initiator 4,4′-azobis (4-cyanovaleric acid) (M_w_ = 280.28 g/mol, ≥98% (T)) (ACVA), dichloromethane (M_w_ = 84.93 g/mol, pro analysis, ≥99.5% (GA)), trimethylamine (M_w_ = 101.19 g/mol, pro analysis, ≥99.5% (GC)), thionyl chloride (M_w_ = 118.97 g/mol, ReagentPlus, ≥99%) and styrene (M_w_ = 104.15 g/mol, ReagentPlus, ≥99%) were provided by Sigma-Aldrich (St. Louis, MO, USA). Chloroform (M_w_ = 119.38 g/mol, pro analysis, ≥99.5%), hexane (M_w_ = 86.18 g/mol, pro analysis, ≥99% (GC)), dimethylformamide (M_w_ = 73.09 g/mol, ACS reagent, ≥99.8%) and acetone (M_w_ = 58.08 g/mol, pro analysis, ≥99.5% (GC)) were provided by Kemika d.d. (Zagreb, Croatia). All chemicals were used as received. Distilled water used for rinsing test samples after each step of chemical modification was prepared according to the ASTM type II specifications, using a TKA MicroMed system (Niederelbert, Germany).

### 2.2. Wood Modification

**Figure 1:**
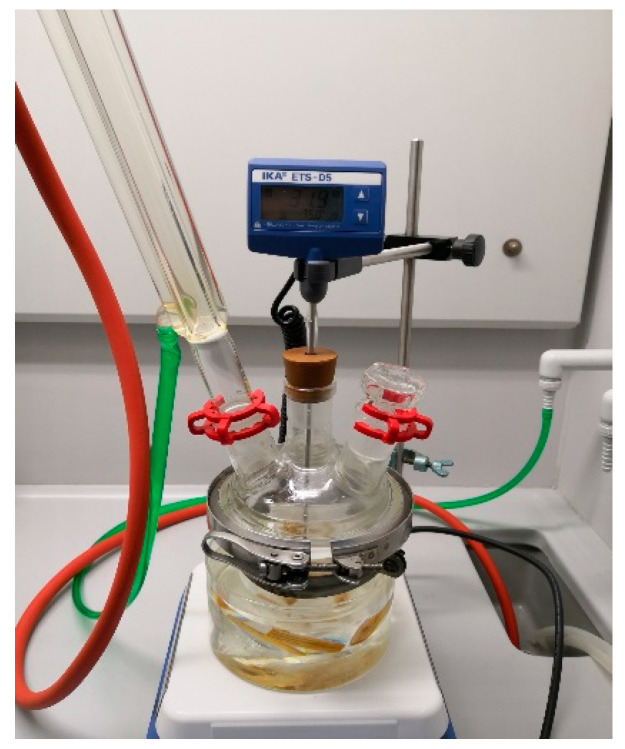
Photograph of polymerisation.

Fir wood (*Abies alba* Mill.) was chemically modified with styrene monomer. RAFT modification was performed on a wooden sample to graft poly (styrene) (PS) onto the wood. Part of the procedure was taken from Cabane et al. [20]. Grafting was carried out using styrene monomer and modified azo initiator 4,4′-azobis (4-cyanopentanoyl chloride (ACPC)). The modified azo initiator 4,4′-azobis(4-cyanopentanoyl chloride) (ACPC) was selected as a polymerisation initiator due to its easy non-catalysed coupling to various surfaces and low decomposition temperature. ACPC was synthesised according to the following procedure: thionyl chloride was slowly added to a cooled solution of 4,4′-azobis (4-cyanopentanoic acid) and triethylamine in dichloromethane (DCM) under nitrogen atmosphere. After 30 min, the ice bath was removed, and the reaction was continued for 12 h at room temperature. After filtration of the precipitate, DCM and traces of unreacted thionyl chloride were removed by rotary evaporation at room temperature. The solid obtained was dissolved in 180 mL of chloroform, then precipitated in 180 mL of hexane, and dried under a high vacuum for 1.5 h at room temperature. After creating the initiator, wood samples were placed under vacuum for 30 min (550 mbar) to extract air. After 30 min, the initiation mixture consisting of 3.02% of initiator (ACPC) and 8.78% triethylamine mixed in 100 mL of dimethylformamide was prepared. The solution was then added to the reactor and stirred with the wood samples for 10 h. After completion of the initiation reaction, wood samples were blotted and washed with acetone to remove unreacted ACPC. For polymerisation, 100 mL of styrene was poured into the reactor, heated to 75 °C and stirred for 2 h (Figure 1).

**Figure 2:**
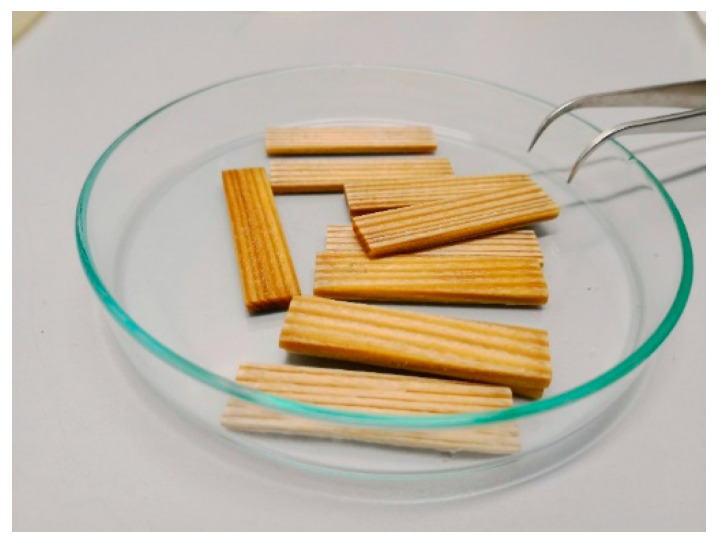
Modified fir wood samples.

After completion of the polymerisation, the wood samples were washed with acetone and distilled water to remove unreacted monomers and free polymer chains (Figure 2). Then, samples were oven-dried for 24 h at 103 °C.

Due to the incorporation of styrene into the cell wall, samples mass increased. Weight percentage gain (WPG) was calculated as the ratio of the mass increase in the treated sample (according to the same sample before modification in the absolute dry state) and the mass of the sample in the absolute dry state. As a result, the average weight percentage gain (WPG) was 18.89%.

### 2.3. Characterisation

#### 2.3.1. FTIR-ATR Spectroscopy

Fourier-transform infrared spectroscopy (FTIR) is a valuable technique for studying differences in chemical alterations that occurred in wood substrates and their main polymer components (cellulose, lignin) after modification. The chemical composition of unmodified and styrene modified fir wood was analysed with an infrared spectrometer Perkin Elmer Spectrum One (Waltham, MA, USA) equipped with an attenuated total reflection (FTIR-ATR) accessory (ZnSe) at room temperature. All spectra were recorded in the range 4000–600 cm^−1^ collecting four scans with a resolution of 4 cm^−1^.

#### 2.3.2. Dynamic Mechanical Analysis (DMA)

Dynamic viscoelastic properties of the modified and unmodified fir wood were measured using a Du Pont 983 Dynamic Mechanical Analyser DMA (TA Instruments, New Castle, DE, USA) interfaced to a 2100 series Thermal Analyst controller. Test samples were measured at the fixed frequency of 1 Hz and temperature ranging from −50 °C to 120 °C, at a heating rate of 3 °C min^−1^. The DMA low mass vertical clamps were used for DMA measurements. Two test samples were analysed, and their dimensions were 25 mm × 10 mm × 2 mm (l × w × t). Storage modulus (E′), loss modulus (E″), and mechanical loss factor (tan δ) were recorded and plotted against temperature. Liquid nitrogen was used to cool the system to sub-ambient temperatures.

#### 2.3.3. Thermogravimetric Analysis (TGA)

The thermal degradation behaviour and thermal stability were evaluated using thermogravimetric analysis (TGA). Thermogravimetric analysis was performed under a nitrogen atmosphere (60 mL/min) using a Q500 TGA analyser (TA Instruments, New Castle, DE, USA) at a heating rate of 10 °C/min from room temperature to 800 °C. Samples of approximately 10 mg were analysed.

## 3. Results and Discussion

### 3.1. FTIR-ATR Spectroscopy

**Figure 3:**
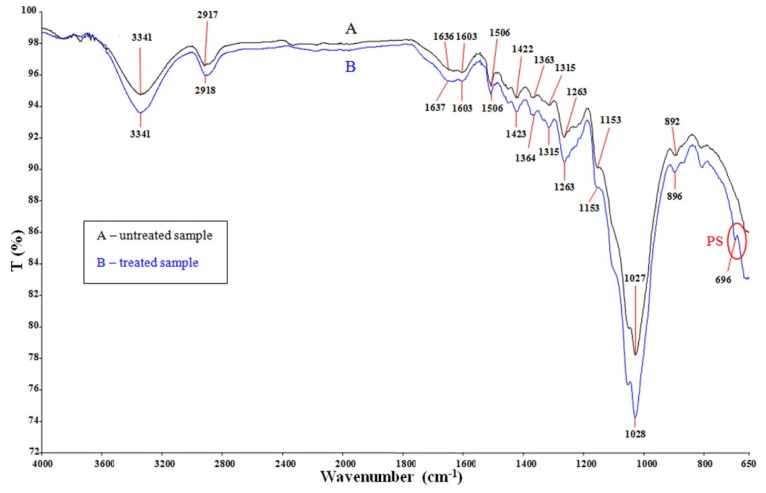
FTIR spectra of unmodified (black curve) and styrene modified (blue curve) fir wood (Sample 1).

In order to confirm the incorporation of styrene in the fir wood and its interaction (chemical reaction), samples were subjected to analysis by FTIR-ATR spectroscopy. FTIR spectra for the unmodified and styrene modified samples of fir wood are presented in Figure 3. From the FTIR spectra, it was found that there is no significant difference between those two spectra qualitatively. The unmodified (A) and modified (B) fir wood show the absorption peak at 3341 cm^−1^ (Sample 1) due to the hydroxyl groups, with the absorption peak around 2917 cm^−1^ corresponding to C-H stretching in cellulose and hemicellulose present in the wood. The absorption peak appearing at 1637 cm^−1^ (Sample 1) is for C=O in hemicelluloses or lignin. Additional lignin-related peaks are those at 1603 cm^−1^ for aromatic skeletal and C=O stretch vibrations, 1506 cm^−1^ for the C=C stretch of aromatic skeletal vibration and 1263 cm^−1^ for the G-ring plus C=O stretch [23].

The amorphous and crystallised cellulose have been characterised using the assigned peak for cellulose at 1422 cm^−1^ and 1364 cm^−1^ for CH_2_ and methoxyl C-H bendings of lignin, respectively, the band at 1315 cm^−1^ (CH_2_ wagging) was used as specified for the crystalline cellulose, 1153 cm^−1^ and 892–896 cm^−1^ for C-O-C stretching and C-H deformation, respectively [23,24]. The most intense peak around 1027 cm^−1^ is related to the C-O stretching of cellulose and hemicellulose. After modification with styrene, a new absorption peak appeared at 696 cm^−1^ due to the phenyl group of styrene. Generally, the polymer will form a chemical bond with the surface or be attached physically. Therefore, the presence of characteristic peaks of styrene indicated the presence of the PS in the cell wall.

### 3.2. Thermomechanical Behaviour of Fir Wood

The DMA measurement consists of observing time-dependent deformation behaviour of a sample under periodic, mostly sinusoidal deformation force with very small amplitudes [25]. The physical and thermomechanical properties of polymeric material strongly depend on its structure, relaxation processes, and morphology. The dynamic viscoelastic property is commonly evaluated by storage modulus E′, loss modulus E″, and mechanical damping tan δ. The storage modulus represents the ability of any material to store mechanical energy and resist deformation [26]. The loss modulus E″ is a measure of the energy dissipated or lost as heat per cycle under deformation and measures the viscous response of the material. The storage modulus could be used to evaluate the rigidity or stiffness of the material, whereas E″, the loss modulus, is a measure of the energy lost as heat. The tan δ gives the balance between the elastic and viscous phases in a polymeric structure (E′/E″ = tan δ). DMA is an advantageous technique due to its ability to differentiate between the single T_g_ of a homogeneous material and the thermal transitions of heterogeneous material. The observation of one or several T_g_ values and the effect of the chemical modification of wood can be related to the wood viscoelastic response.

**Figure 4:**
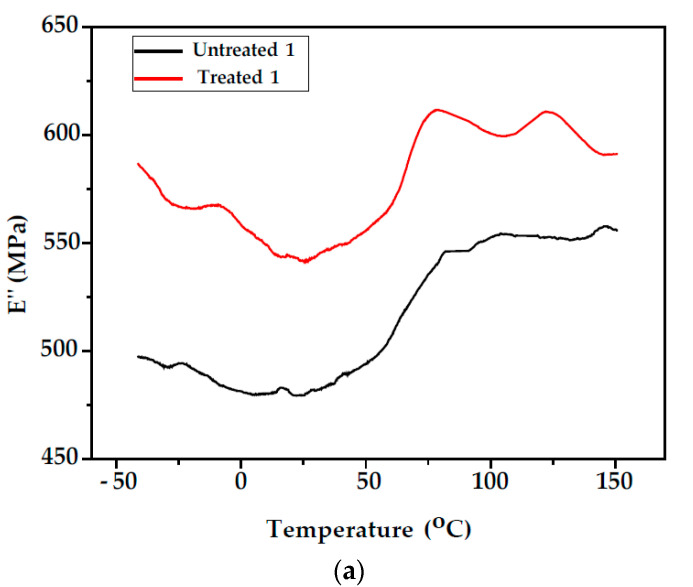

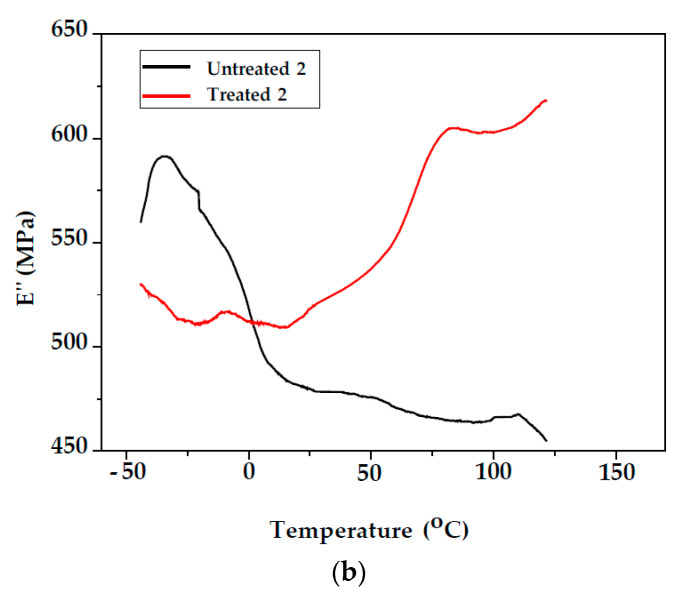
Temperature dependencies of loss modulus, E″, of unmodified and modified fir wood (**a**) Sample 1; (**b**) Sample 2.

Figure 4 shows the variation in loss modulus E″ as a function of the temperature for unmodified and modified fir wood test specimens (Samples 1 and 2). The obtained results are reported in Table 1. The α transition is attributed to the initiation of micro-Brownian motion of large segments of polymeric chains in wood and corresponds to the T_g_. Above that temperature, the material becomes soft and flexible and is either an elastomer or a highly viscous liquid. From the E″ vs. T plot, one T_g_ for (unmodified wood) was observed at 93.1 °C (Sample 1) and at 89.6 °C (Sample 2). Olsson and Salmen (1997) determined how this transition temperature correlates with the molecular motion of lignin, essentially proving it to be the lignin T_g_ [27,28]. Two separate T_g_ were observed for modified fir wood. The first transition attributed to T_g_ of lignin component and a second transition at higher temperatures (125 °C) attributed to T_g_ of polystyrene. The T_g_ values for unmodified and modified fir wood samples (Samples 1 and 2) are presented in Table 1. The T_g_ value of lignin for modified wood was slightly shifted to a lower temperature. This suggests that the amorphous phase content was increased with the addition of amorphous PS. The T_g_ values of PS were observed at almost the same temperature location.

**Figure 5:**
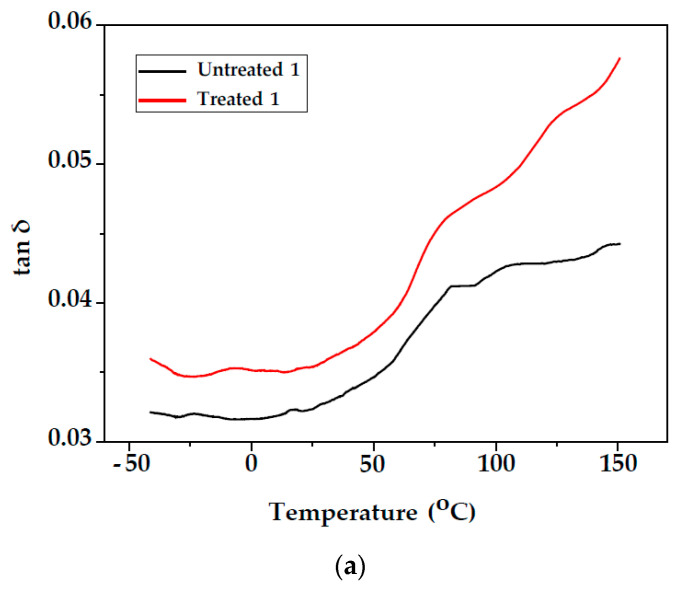

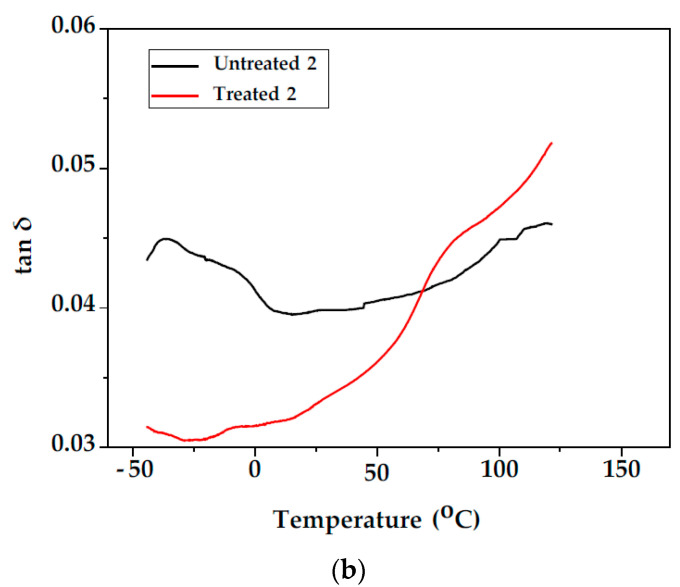
Temperature dependencies of tan δ, of unmodified and modified fir wood (**a**) Sample 1; (**b**) Sample 2.

The variation in tan δ as a function of the temperature for unmodified and modified fir wood test specimens (Samples 1 and 2) are presented in Figure 5. The maximum value of tan δ peak corresponds to the T_g_ value of lignin and for modified samples are lower than those for the unmodified samples, as seen in Table 1. The T_g_ values for the unmodified samples obtained from the loss modulus peaks E″ were lower than those obtained from the tan δ peaks. The T_g_ values of PS from E″ are slightly higher than from the tan δ values.

**Figure 6:**
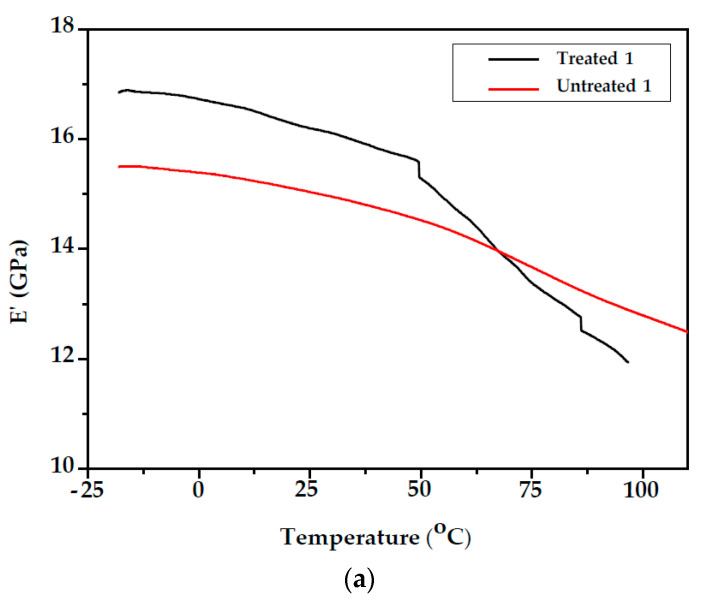

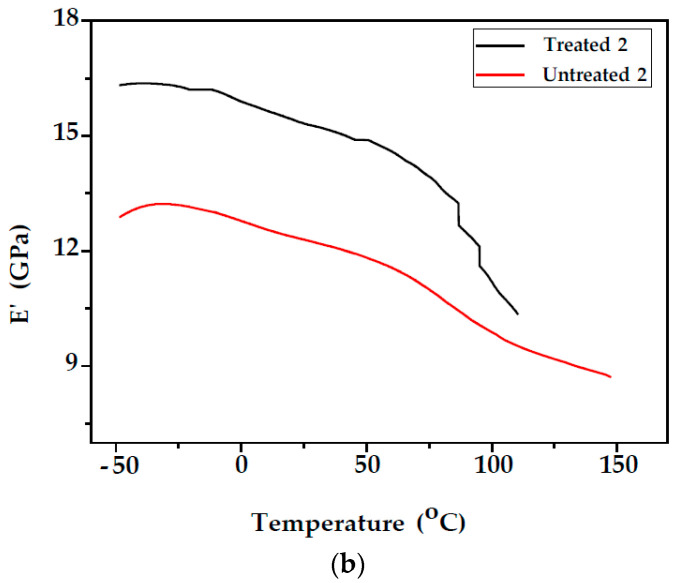
Temperature dependencies of storage modulus, E′, of unmodified and modified fir wood (**a**) Sample 1; (**b**) Sample 2.

The variation in the storage modulus E′ of unmodified and modified fir wood samples (Samples 1 and 2) as a function of temperature can be observed in Figure 6. From the E′ vs. T curves, it can be observed that the storage modulus for unmodified and modified fir wood of Samples 1 and 2 decreased with increasing temperature. This behaviour can be attributed to the increase in the chain mobility of the polymeric components of the wood cell walls at higher temperatures [29]. As a result, the drop in the modulus on passing through the T_g_ is higher for the modified samples compared with the unmodified. After styrene treatment, storage modulus E′ increased compared with the unmodified wood in both samples, which may be due to the increase in the stiffness (Table 1). Since the DMA instrument cannot control and regulate humidity, testing requires that the wood is in two possible states: either completely plasticised or oven-dry [30].

### 3.3. Thermal Degradation Behaviour of Fir Wood

Wood in an inert atmosphere undergoes a complex thermal degradation process, which is greatly affected by its three main components: hemicellulose, cellulose, and lignin.

**Figure 7:**
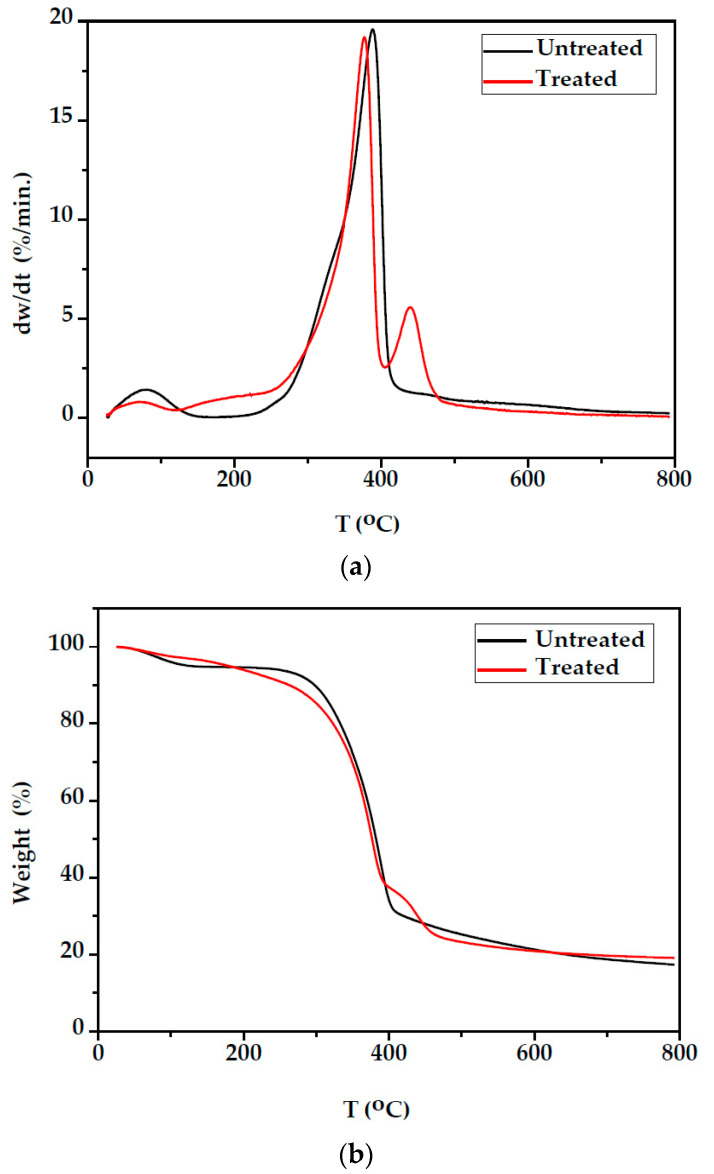
DTG (**a**) and TG (**b**) curves of unmodified and modified fir wood.

The derivative thermogravimetric (DTG) and thermogravimetric (TG) curves for both the unmodified and modified fir wood under a nitrogen environment are shown in Figure 7. Table 2 shows the corresponding thermal characteristics for both unmodified and modified wood samples.

Figure 7 illustrates the thermogravimetric (TG/DTG) curves of unmodified fir wood under a nitrogen environment. Three-weight loos steps were observed. The first step of degradation occurred between 25 °C to 150 °C with T_max_ at 72.2 °C was attributed to moisture loss and absorbed water. Weight loss during this period was 5.2%. The initial decomposition temperature at 289.3 °C shows the starting point of wood decomposition. Typically, wood has a larger shoulder region due to the degradation of hemicelluloses before the degradation of cellulose. According to the DTG thermogram of unmodified fir wood, two peaks observed in the interval temperature from 180 to 425 °C correspond to the decomposition of hemicellulose and cellulose. Hemicellulose decomposition was observed from 180 to 340 °C as a less pronounced shoulder at 322 °C and cellulose decomposition from 340 to 425 °C with a maximum peak at 379.9 °C. Cellulose is highly crystalline, whereas hemicellulose is amorphous and degrades before cellulose. According to Chow and Pickeles, this weight loss involves decomposing cellulose crystals and hemicellulose into levoglucosan [31]. Lignin was the most difficult one to decompose because its decomposition is slow and occurs from 200 to 900 °C [32]. Therefore, a shoulder is observed at about 420 °C with a peak at 458 °C. This high-temperature shoulder can be assigned to the end of an overlapping degradation process of lignin. According to the DTG thermogram of the modified fir wood, the weight loss occurs at three distinct steps. The first step at 70.1 °C (T_max_^1^) with 2.783% weight loss was a dehydration process, in which free water in the wood sample evaporated. The mass loss of modified fir wood samples was lower than untreated samples from 25 to 150 °C, indicating that the moisture content of modified fir wood was lower than that of the untreated wood. The second step was at 377.0 °C (T_max_^2^) with 54.37% weight loss mainly caused by the thermal decomposition of cellulose [31].

The third weight loss at the temperature range from 400 to 490 °C with peak temperature at 440.3 °C was associated with the degradation of polystyrene. It can be seen from Table 2 that for the modified wood sample, the temperature values corresponding to the maximum decomposition rate (T_max_^1^ and T_max_^2^) decreased compared with the unmodified fir wood. The initial decomposition temperature (T_i_) in the modified fir wood sample was slightly higher than the unmodified wood sample. This result indicated that the incorporation of styrene improved the thermal stability of the fir wood. Table 2 shows the temperatures corresponding to the end of decomposition (T_f_). It was observed that the T_f_ value of the modified fir wood sample was higher for 23 °C compared with the unmodified sample. The residual at 800 °C of the modified wood sample is observed to be higher (19.27%) than the unmodified fir wood sample. This residue can be charcoal from lignin decomposition.

## 4. Conclusions

Testing the mechanical properties of wood with a dynamic mechanical analyser, we obtained a significantly better understanding of the organisation and properties of polymers. It is important to ensure maximum uniform samples at each stage of sample preparation (approximately equally wide rings and equal share of late and earlywood zone).

Infrared data showed that new absorption appears at 700 cm^−1^ due to the phenyl group of styrene. The presence of a characteristic peak of styrene indicated the successful impregnation of this monomer into the composites. DMA results showed that the modified wood caused an increase in the glass transition temperature relative to the unmodified wood. The thermal stability of unmodified and modified wood samples was evaluated by thermogravimetric analysis (TGA).

The polymeric materials tested for DMA are mostly homogeneous structures. At the same time, wood is known for its inhomogeneity, and samples must be taken that are approximately equal zones of early and late wood and free of bumps and wood defects.

This preliminary study attempted to set parameters for further investigations, and a sound basis was made for further process optimisation.

## Figures and Tables

**Table 1:** Results of the dynamic mechanical analysis on unmodified and modified fir wood samples.

Fir Wood	Temperature (°C)	Amplitude (mm)	E′ (GPa)	T_g from E″_ of Wood (°C)	T_g from tan δ_ of Wood (°C)	T_g from E__″_ PS (°C)	T_g from tan δ_ PS (°C)
Unmodified							
			E′ initial = 15.5				
Sample 1	−50 to 150	0.3	E′ final = 11.5	93.1	107.4	-	-
			E′ (40 °C) = 14.8				
			E′ initial = 13.0				
Sample 2	−50 to 150	0.4	E′ final = 8.7	89.6	104.9	-	-
			E′ (40 °C) = 12.0				
Modified							
			E′ initial = 16.3				
Sample 1	−50 to 150	0.4	E′ final = 10.3	80.6	77.9	123.8	124.4
			E′ (40 °C) = 15.0				
			E′ initial = 16.8				
Sample 2	−50 to 150	0.3	E′ final = 12.0	83.7	79.8	125.0	125.2
			E′ (40 °C) = 15.2				

**Table 2:** TGA results of the unmodified and modified fir wood.

Fir Wood	T_i_ (°C)	T_f_ (°C)	T_max_^1^ (°C)	T_max_^2^ (°C)	T_max_^3^ (°C)	Residue at 800 °C (%)
Unmodified	289.3	400.4	72.2	379.9	-	17.27
Modified	312.3	423.5	70.1	377.0	440.3	19.13

T_i_—initial decomposition temperature; T_f_—final decomposition temperature; T_max_—temperature of a maximum rate of weight loss.

## Data Availability

The data presented in this study are available upon request from the corresponding author.
